# Inhibition of the Mitochondrial Enzyme ABAD Restores the Amyloid-β-Mediated Deregulation of Estradiol

**DOI:** 10.1371/journal.pone.0028887

**Published:** 2011-12-12

**Authors:** Yun-An Lim, Amandine Grimm, Maria Giese, Ayikoe Guy Mensah-Nyagan, J. Ernest Villafranca, Lars M. Ittner, Anne Eckert, Jürgen Götz

**Affiliations:** 1 Alzheimer's & Parkinson's Disease Laboratory, Brain & Mind Research Institute, University of Sydney, Camperdown, New South Wales, Australia; 2 Neurobiology Laboratory, Psychiatric University Clinics Basel, University of Basel, Basel, Switzerland; 3 Equipe Steroïdes, Neuromodulateurs et Neuropathologies, Université de Strasbourg, Strasbourg, France; 4 Villafranca Consulting, San Diego, California, United States of America; Massachusetts General Hospital and Harvard Medical School, United States of America

## Abstract

Alzheimer's disease (AD) is a conformational disease that is characterized by amyloid-β (Aβ) deposition in the brain. Aβ exerts its toxicity in part by receptor-mediated interactions that cause down-stream protein misfolding and aggregation, as well as mitochondrial dysfunction. Recent reports indicate that Aβ may also interact directly with intracellular proteins such as the mitochondrial enzyme ABAD (Aβ binding alcohol dehydrogenase) in executing its toxic effects. Mitochondrial dysfunction occurs early in AD, and Aβ's toxicity is in part mediated by inhibition of ABAD as shown previously with an ABAD decoy peptide. Here, we employed AG18051, a novel small ABAD-specific compound inhibitor, to investigate the role of ABAD in Aβ toxicity. Using SH-SY5Y neuroblastoma cells, we found that AG18051 partially blocked the Aβ-ABAD interaction in a pull-down assay while it also prevented the Aβ42-induced down-regulation of ABAD activity, as measured by levels of estradiol, a known hormone and product of ABAD activity. Furthermore, AG18051 is protective against Aβ42 toxicity, as measured by LDH release and MTT absorbance. Specifically, AG18051 reduced Aβ42-induced impairment of mitochondrial respiration and oxidative stress as shown by reduced ROS (reactive oxygen species) levels. Guided by our previous finding of shared aspects of the toxicity of Aβ and human amylin (HA), with the latter forming aggregates in Type 2 diabetes mellitus (T2DM) pancreas, we determined whether AG18051 would also confer protection from HA toxicity. We found that the inhibitor conferred only partial protection from HA toxicity indicating distinct pathomechanisms of the two amyloidogenic agents. Taken together, our results present the inhibition of ABAD by compounds such as AG18051 as a promising therapeutic strategy for the prevention and treatment of AD, and suggest levels of estradiol as a suitable read-out.

## Introduction

In the Alzheimer's disease (AD) brain, amyloid-β (Aβ) has a central yet only partly understood role in the neurodegenerative process [1]. Apart from constituting the amyloid plaque as a classical hallmark lesion of AD, Aβ acts via a plethora of pathways to induce synaptic and neuronal degeneration [2]–[4]. Many studies reveal that in exerting its toxicity, Aβ binds to specific receptors and/or lipids at the neuronal cell membrane, and some studies even suggest a disruption of ion homeostasis by forming channels or pores [5], [6]. To better understand what the prerequisites are for Aβ toxicity, we and others used transgenic mouse models and found that Aβ mediates its toxicity in part through the NMDA receptor, with an essential role for the microtubule-associated protein tau [7]–[9], that similar to Aβ, also forms insoluble aggregates in the AD brain. Over-activation of the NMDA receptor complex results in excessive nitric oxide (NO) levels, causing down-stream protein misfolding and aggregation, as well as mitochondrial dysfunction. The toxic signaling pathway further involves the release of mitochondrial cytochrome c and the activation of down-stream caspases as well as the formation of ROS (reactive oxygen species) [10]–[12], highlighting mitochondria as a prime down-stream target of Aβ [13]–[15].

Interestingly, mitochondria represent not only an indirect target; instead, in several studies Aβ has been localized to [16] and shown to act directly on mitochondria [17], [18] whose function it impairs [19]–[22]. Among the mitochondrial proteins to which Aβ has been shown to bind is the enzyme amyloid-binding alcohol dehydrogenase (ABAD) [23], [24]. ABAD interacts with Aβ and is a major determinant of Aβ toxicity [17], [25], [26]. Specifically, in mice doubly transgenic for ABAD and the Aβ-precursor APP, the toxic effects of Aβ are aggravated compared to what is found in APP single transgenic mice [17].

ABAD is the Type 10 member of a protein family, known as 17β-hydroxysteroid dehydrogenases (HSD17B) [27]. The enzyme is found in mitochondria, while the other known fourteen family members are confined to the endoplasmic reticulum (ER) suggesting that ABAD has a specialized function within mitochondria [28]. ABAD converts estradiol to estrone [29], and its levels are critical as optimal estradiol levels are an important determinant of neuronal survival [29]. In post-menopausal women, the estrogen replacement therapy has been shown to delay the onset of AD [30]. In the placenta and in ovaries, ABAD inactivates estradiol by oxidizing it to estrone [31], [32], and this may also occurs in testis [33]. Interestingly, ABAD levels themselves are sensitive to estradiol levels suggesting a feedback loop in the regulation of its activity [34].

The many reports of ABAD's enzymatic action on various substrates *in vivo* have been challenged, however, by strong evidence that a catalytically inactive mutant of ABAD as identified in a young boy had no ill effects on his health [35]. In addition, ABAD was found to be one of only three proteins that comprise the fully functional mammalian mitochondrial RNAse P [36], a function that may not require enzymatic activity and that links ABAD directly to the production of mitochondrial electron transport chain proteins and reactive oxygen species (ROS) generation [37].

Binding of Aβ to ABAD induces a conformational change that is inhibited by NAD^+^ (nicotinamide adenine dinucleotide), with binding of Aβ and NAD^+^ being mutually exclusive [38]. Aβ binding results in the loss of ABAD function and ultimately, neuronal apoptosis [39], [40]. To directly determine whether Aβ-induced toxicity is mediated by ABAD inhibition and to establish estradiol levels as a suitable readout, we here employed the use of AG18051, a novel ABAD inhibitor [41].

## Materials and Methods

### Cell culture and incubation with amyloid peptides

SH-SY5Y neuroblastoma cells (DSMZ, Braunschweig, Germany; DSMZ No. ACC 209) were grown in Dulbecco's Modified Eagle Medium: F-12 (DMEM: F-12) supplemented with 2 mM L-glutamine, 1% penicillin/streptomycin and 10% fetal bovine serum (FBS) (GIBCO, Basel, Switzerland) [42], [43]. Aβ42, human amylin (HA), biotinylated Aβ42 and biotinylated HA were purchased from Bachem (Germany) (H-1368, H-7905, H-5642 and 3004028, respectively). The negative control, biotin, was purchased from Sigma (B4639). Biotinylated and unmodified Aβ42 were dissolved in DMSO to make stocks of 5 mM and stored at −80°C until use. Biotinylated and unmodified HA were dissolved in 0.01 M acetic acid (AA) to make stocks of 5 mM and also stored at −80°C until use. Biotin was dissolved in DMSO to make stocks of 5 mM and kept at −80°C until use. Aging of the peptides was induced by shaking at 1000 rpm for 4 days at 37°C. 0.5 µM Aβ42 or human amylin (HA) was used for all experiments in this study, while the treatment duration was always 5 days. Pre-treatment experiments were performed by incubating SH-SY5Y cells with 0.05 µM AG18051 for 24 hours, washing 3 times with warm PBS, and then treating the cells with 0.5 µM Aβ42 or HA for 5 days. Co-treatment experiments were done by incubating SH-SY5Y cells for 5 days with 0.05 µM AG18051 and 0.5 µM Aβ42 or HA, respectively.

### LDH and MTT assays

LDH and MTT assays were chosen to provide indications for cell viability after treatments. Assays were obtained from Roche and were performed according to the manufacturer's protocols. Briefly, cells were exposed to the various treatments, after which the medium was retrieved for LDH analysis, while the remaining cells were washed 3 times with sterile PBS and the MTT assay performed with the cells.

### Pull-down assay

SH-SY5Y cells were grown to 70% confluency and treated with vehicle, biotinylated Aβ42, biotinylated HA, or biotin, respectively, at a final concentration of 0.5 µM for 5 days. In addition, 0.5 µM biotin and 0.5 µM biotinylated Aβ42, respectively, was co-incubated with 0.05 µM AG18051 for 5 days. After 5 days, cells were once washed with pre-warmed PBS and immediately scraped with 500 µL ice-cold IP buffer (10 mM Tris, 0.1 M NaCl, 1 mM EDTA), supplemented with the Complete EDTA-free protease inhibitor cocktail (1∶25) (Roche, Basel, Switzerland), and spun at 14 000× g for 10 minutes at 4°C. 20 µl of magnetic Dynabeads MyOne™ Streptavidin T1 (Invitrogen) were added to each tube and tubes were rotated for 30 minutes at room temperature. Beads were subsequently washed 3 times with 1× PBS, followed by boiling in loading buffer containing β-mercaptoethanol at 95°C for 5 minutes at 1000 rpm to release bound peptides. Samples were then briefly spun down at room temperature and supernatants loaded onto 12% glycine gels for electrophoresis and Western blotting.

### Quantification of estradiol as a functional read-out of ABAD activity

After treatment with amyloid peptides and controls, the cell culture medium was collected and cells were resuspended in cell lysis buffer consisting of 150 mM Tris HCl, 150 mM NaCl, 1% NP-40, 0.1% SDS, 2 mM EDTA and Complete protease inhibitor (1∶50) (Roche Diagnostics). The estradiol assay was performed according to the manufacturer's guidelines (Estradiol EIA Kit, Cayman). In brief, the plate was loaded with samples, along with the estradiol tracer and the specific antiserum to estradiol and incubated for one hour at room temperature. After five washing steps, Ellman's Reagent was added and the plate developed for 60 minutes with gentle shaking at room temperature. The calculated estradiol concentration was normalized to the total protein content of the samples.

### Mitochondrial respiration in vital cells

After treatment with amyloid peptides and controls, mitochondrial oxygen consumption was measured at 37°C using an Oroboros Oxygraph-2k system. Five million cells were added to 2 ml of a mitochondrial respiration medium containing 0.5 mM EGTA, 3 mM MgCl_2_, 60 mM K-lactobionate, 20 mM taurine, 10 mM KH_2_P0_4_, 20 mM HEPES, 110 mM sucrose and 1 g/l BSA (pH 7.1). To measure the state 4 respiration of complex I, 5 mM pyruvate and 2 mM malate were added and cells permeabilised with 15 µg/ml digitonin. Afterwards, 2 mM ADP was added to measure state 3 respiration. The integrity of the mitochondrial membrane was checked by the addition of 10 µM cytochrome c. After determining coupled respiration, 0.4 µM FCCP (Carbonyl cyanide p-(trifluoro-methoxy) phenyl-hydrazone) was added and respiration was measured in the absence of a proton gradient. To inhibit complex I activity, 0.5 µM rotenone was added.

### Determining ROS levels

Levels of ROS were measured using different fluorescent probes that allow detection at different cellular sites. The non-fluorescent probe 2′,7′-dichlorodihydrofluorescein diacetate (H2DCF-DA) was used to measure cytosolic ROS, most notably hydrogen peroxide [44]. To determine levels of superoxide anion radicals, DHE was used, which is oxidized to the fluorescent ethidium cation by O2^.−^ [22]. For detection of mitochondria-associated ROS we used the probe dihydrorhodamine (DHR), which localizes to mitochondria and when oxidized by ROS, particularly peroxinitrite, fluoresces to the positively charged rhodamine 123 derivate. Treated cells (Aβ42, HA, and vehicle) were loaded with 10 µM H2DCF-DA, 10 µM DHR or 10 µM DHE, respectively, for 15 min. After washing twice with Hank's balanced salt (HBS) solution, the formation of fluorescent products was measured by detecting the emitted fluorescent units per 5×10^5^ cells using the Fluoraskan Ascent FL multiplate reader (Labsystems, Helsinki, Finland) (i) at 485 nm (excitation)/538 nm (emission) for both dichlorofluorescein (DCF) generated from H2DCF-DA via oxidation, and DHR, and (ii) at 530 nm (excitation)/590 nm (emission) for DHE, as previously described [45].

## Results

### Aβ42 binds to and down-regulates ABAD activity, different from human amylin

**Figure 1:**
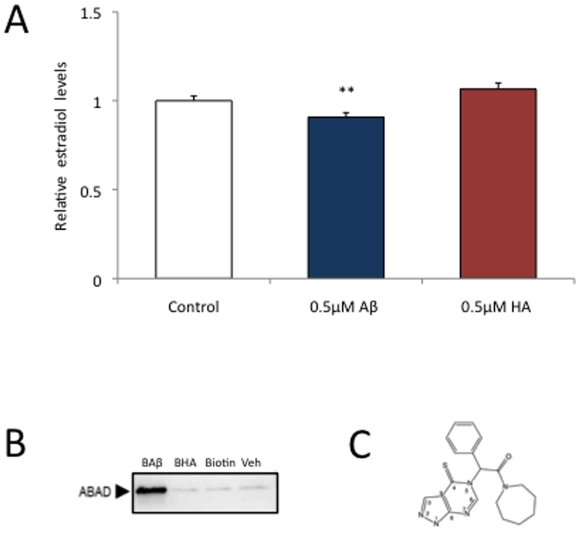
Aβ42 binds to and impairs ABAD activity, while HA (human amylin) does not. (A) Treatment of SH-SY5Y human neuroblastoma cells with Aβ42 causes decreased levels of estradiol, indicative of an impairment of ABAD activity, while HA does not. Results are means ± SE, (n = 5 to 6 per group), **, *P*<0.01 (B) Pull-down of ABAD from SH-SY5Y cells shows that different from HA, Aβ42 can bind to ABAD *in vitro*. (C) Structure of the ABAD inhibitor, AG18051 (adapted from Kissinger et al., JMB 2004).

Aβ exerts its toxicity in part by impairing ABAD [17], an enzyme known to convert estrone to estradiol. To determine the toxicity of the major fibrillogenic form of Aβ and the role ABAD has in this process, we incubated human SH-SY5Y neuroblastoma cells for 5 days with Aβ42, followed by measuring estradiol levels in the cell lysate. We found that its levels were significantly decreased after Aβ42 exposure (p<0.0001) (Fig. 1A). To determine if the deregulation of ABAD activity is a common phenomenon shared by Aβ with other amyloidogenic proteins, we treated the cells also with human amylin (HA), a protein twice the size of Aβ and known to form aggregates in another disease with protein aggregation, Type 2 diabetes mellitus (T2DM). However, different from Aβ42, HA did not cause reductions in estradiol levels (p<0.0001) (Fig. 1A).

Data obtained by x-ray crystallography of Aβ42-ABAD complexes indicate that it may be the direct association of Aβ42 and ABAD that inhibits ABAD activity, as an association of Aβ with ABAD prevents it from binding to its physiological substrate, NAD^+^ [17]. Since HA, different from Aβ42, did not down-regulate ABAD activity as measured by estradiol levels, and as it is known that Aβ42 is capable of inhibiting ABAD activity by direct interaction [38], we sought to determine whether HA, similar to Aβ, would interact with ABAD. Therefore, SH-SY5Y cells were incubated with 0.5 µM biotinylated Aβ42 (BAβ), biotinylated HA (BHA), biotin or vehicle for 5 days. We found by pull-down that while biotinylated Aβ42 was bound to ABAD, biotinylated HA, along with the negative control, was not (Fig. 1B). Together with the estradiol activity assay, this suggests that the reductions in estradiol levels caused by Aβ42 may be mediated by a direct interaction of Aβ with ABAD.

### The ABAD inhibitor AG18051 causes reduced estradiol levels

**Figure 2:**
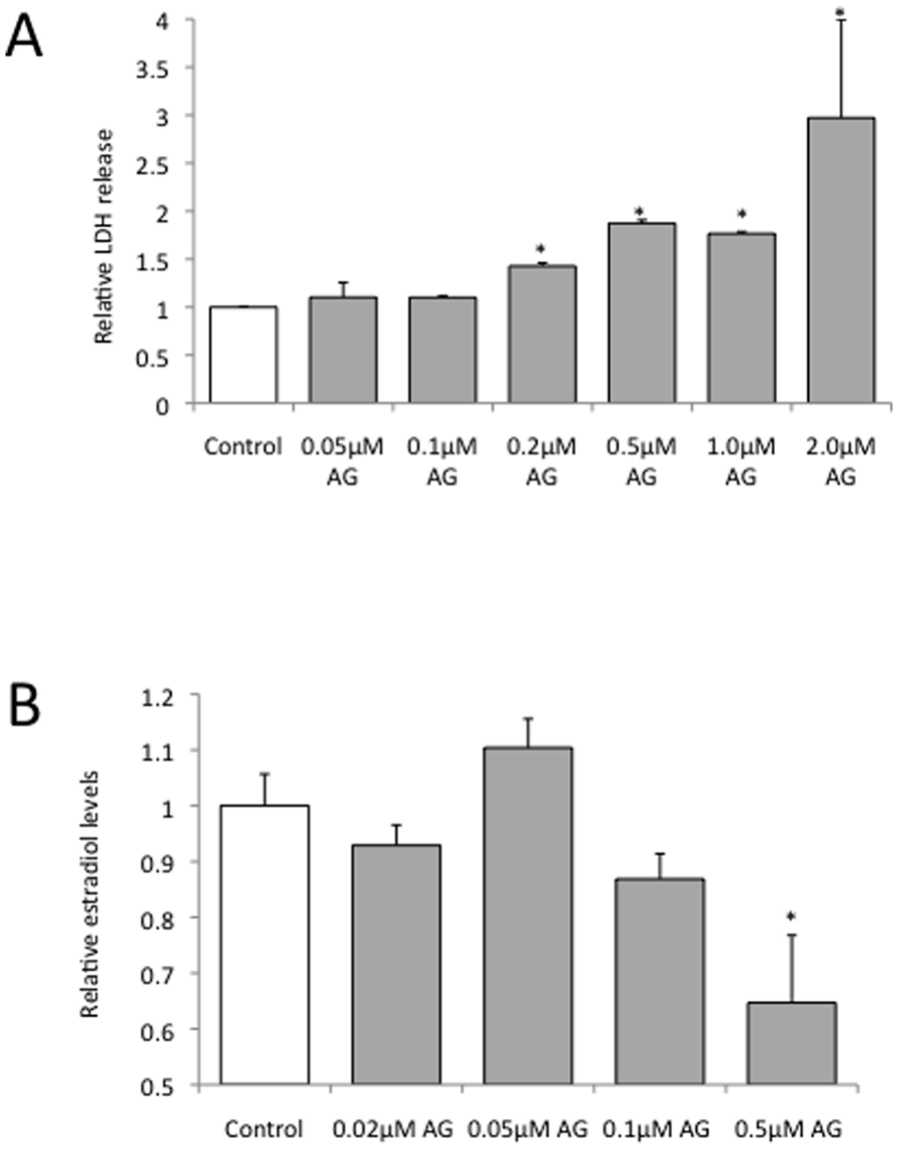
Effects of the ABAD inhibitor AG18051 on cell viability and estradiol levels. (A) LDH assay of SH-SY5Y human neuroblastoma cells incubated with increasing concentrations of AG18051 (normalized to 1 for control) shows that the ABAD inhibitor is not toxic at concentrations of 0.1 µM and below. (B) Treatment of SH-SY5Y cells with increasing concentrations of AG18051 causes reduced levels of estradiol. *, *P*<0.05.

To better understand the effect of Aβ on estradiol levels we used AG18051, a small molecule inhibitor of ABAD with high affinity [41], [46] (Fig. 1C). AG18051 exerts its inhibitory effect by occupying the substrate-binding site of ABAD, which results in the formation of a covalent adduct with the NAD^+^ cofactor. As the interaction of ABAD with NAD^+^ is necessary for its activity, disrupting ABAD/NAD^+^ complex formation obliterates ABAD activity, resulting in decreased estradiol levels. We first determined the toxicity profile of AG18051 in our SH-SY5Y cell culture system. Concentrations of AG18051 up to 0.1 µM were not overtly toxic and therefore, concentrations within the 0.05–0.1 µM range were chosen for subsequent studies (Fig. 2A). The toxicity at higher concentrations argues for a proper dosing. The concentration range applied by us is consistent with the IC_50_ determined for AG18051 in a previous study [47], [48]. With increasing concentrations of the inhibitor, as expected, estradiol levels were reduced (Fig. 2B). At 0.05 µM however, there was a slight, but non-significant increase in estradiol levels suggesting compensatory mechanisms.

### AG18051 prevents Aβ42-induced reductions in estradiol levels

**Figure 3:**
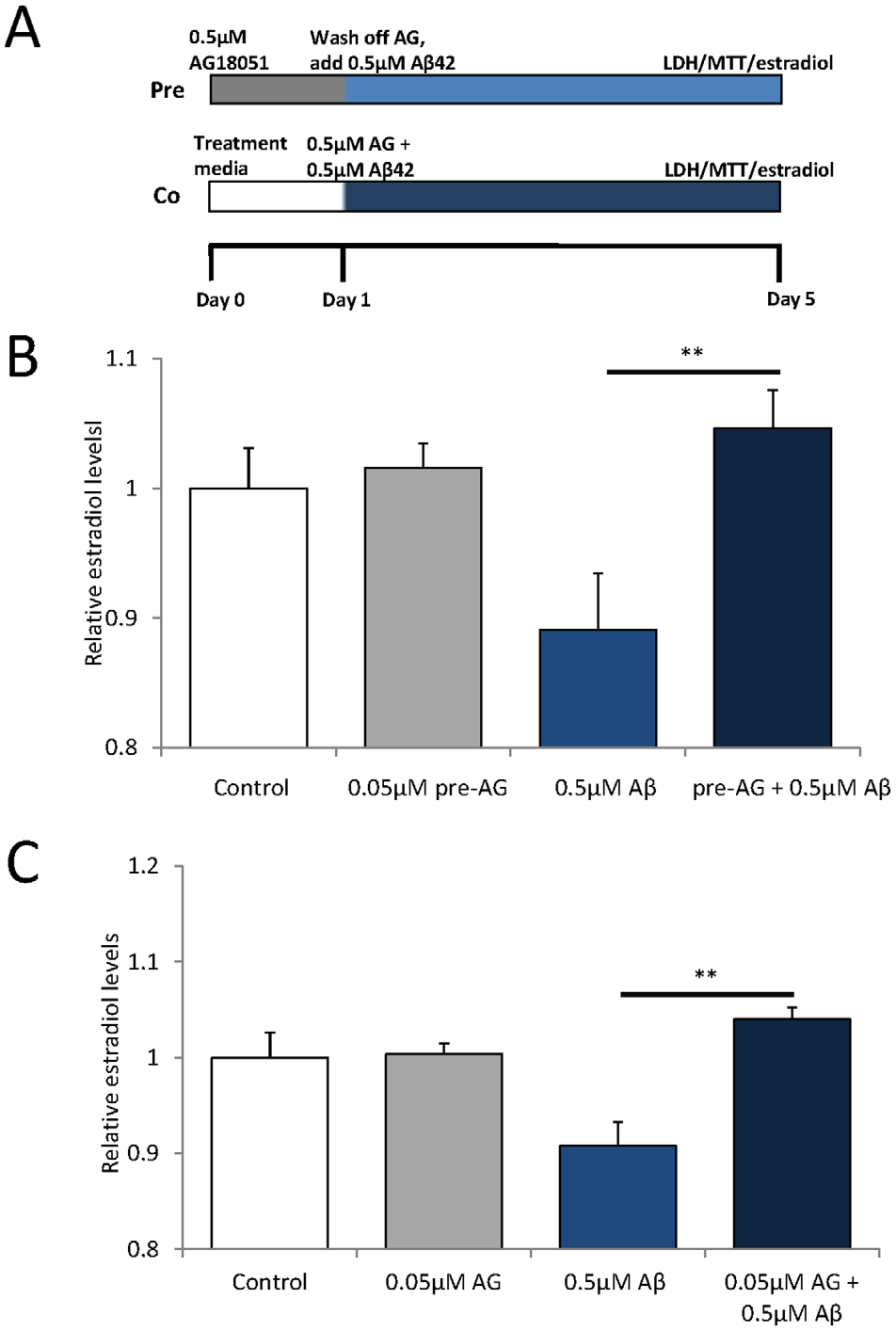
The Aβ-mediated decrease in estradiol levels is prevented by AG18051. (A) Scheme of pre- and co-incubation treatment. (B) Pre-treatment of cells with AG18051 for 24 hours prior to adding Aβ42 maintains estradiol levels compared to the vehicle control, (C) as does co-treatment. **, *P*<0.01.

To determine whether AG18051 has an effect on the Aβ42-induced reduction of estradiol levels (Fig. 1A) and whether the putative neuroprotective effect is via a ‘priming effect’ of the cells, we both pre- and co-incubated SH-SY5Y cells with Aβ42 and 0.05–0.1 µM AG18051 as outlined in the scheme (Fig. 3A). For both treatment conditions, we found that this maintained estradiol levels in the lysate, compared to a significant reduction in the Aβ42-only treatment (Fig. 3B,C). This suggests that AG18051 is neuroprotective, and that it may exert its neuroprotective effect either by priming cells to become resistant to the effects of Aβ42, or via a direct inhibition of the Aβ42-ABAD interaction as previously suggested [41].

### AG18051 partially prevents ABAD-Aβ42 interaction

**Figure 4:**
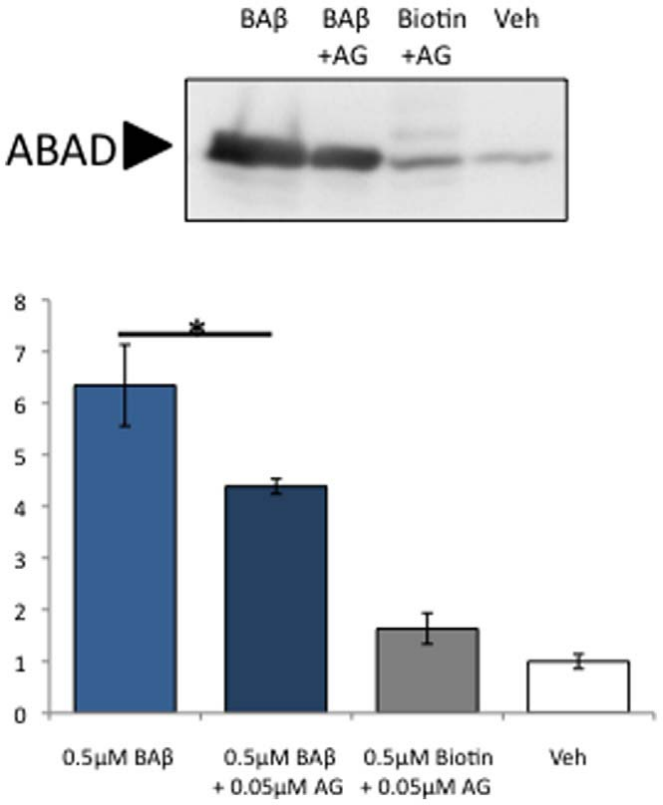
Pull-down of ABAD from SH-SY5Y cells shows that different from HA, Aβ42 can bind to ABAD *in vitro*. The presence of AG18051 significantly decreases the amount of ABAD pulled down by biotinylated Aβ42 (BAβ). *, p<0.05.

To determine whether AG18051 treatment reverses the Aβ42-induced toxicity because AG18051 directly blocks the physical interaction between Aβ42 and ABAD, SH-SY5Y cells were treated with 0.5 µM of biotinylated Aβ42 (BAβ) in the presence of 0.05 µM AG18051. We found that co-incubation of AG18051 significantly decreased the amount of ABAD pulled down by biotinylated Aβ42 (BAβ) (**Fig. 4**). This suggests that AG18051 may prevent Aβ42 toxicity by directly inhibiting the association of Aβ42 with ABAD. In addition, this also suggests that AG18051 may exert its neuroprotective effects via additional pathways other than a direct inhibition of the Aβ42-ABAD interaction.

### Aβ42-mediated reduction in cell viability prevented by ABAD inhibitor AG18051

**Figure 5:**
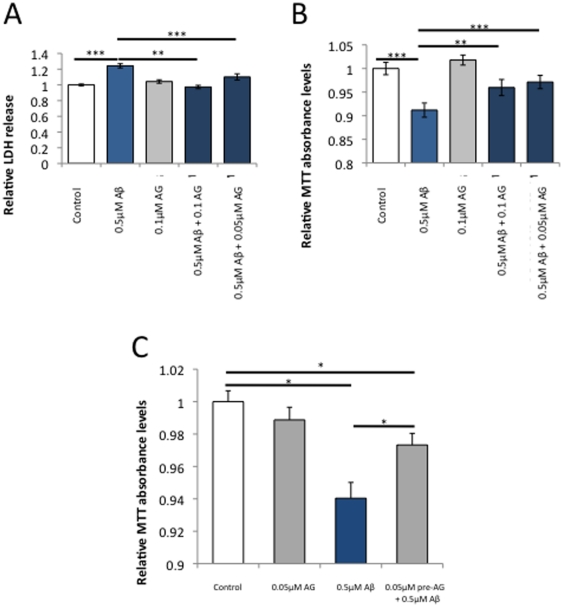
The ABAD inhibitor AG18051 prevents the toxicity and metabolic impairment caused by Aβ. (A) Co-incubation of AG18051 and Aβ42 maintains the Aβ42-induced change in LDH levels at baseline levels. (B) The metabolic impairment as determined with the MTT assay is also prevented by co-incubation of Aβ42 with AG18051. (C) Pre-incubation of the cells with AG18051 for 24 hours prior to adding Aβ42 is similarly protective to Aβ's toxicity as measured with the MTT assay. *, *P*<0.05; **, *P*<0.01; ***, *P*<0.001.

Having determined 0.05–0.1 µM as a suitable concentration range for AG18051, we tested its putative neuroprotective effect in Aβ42 toxicity. SH-SY5Y cells were incubated with 0.5 µM Aβ42 with or without AG18051, and toxicity was determined as increased levels of LDH compared to vehicle control. Co-incubation of Aβ42 with either 0.05 or 0.1 µM AG18051, respectively, resulted in a significant decrease in LDH levels back to control levels (Fig. 5A). This indicates that inhibiting ABAD activity protects from Aβ toxicity.

We next investigated the effect of Aβ on metabolic functions and found that besides significantly increasing LDH levels, 0.5 µM Aβ42 also caused a significant decrease in MTT absorbance, suggesting impaired metabolic functions caused by this amyloidogenic peptide (Fig. 5B). Co-incubation of Aβ42 with either 0.05 or 0.1 µM AG18051, respectively, resulted in a smaller decrease of MTT absorbance (Fig. 5B,C). Since MTT measurements are an indicator of mitochondrial health, the results suggest that Aβ42 induces cell toxicity, as reflected by LDH levels, at least in parts via impaired mitochondrial functions, with inhibition of the mitochondrial enzyme ABAD providing partial protection.

### AG18051 restored Aβ42-induced deficits on oxidative phosphorylation (OXPHOS) capacity

**Figure 6:**
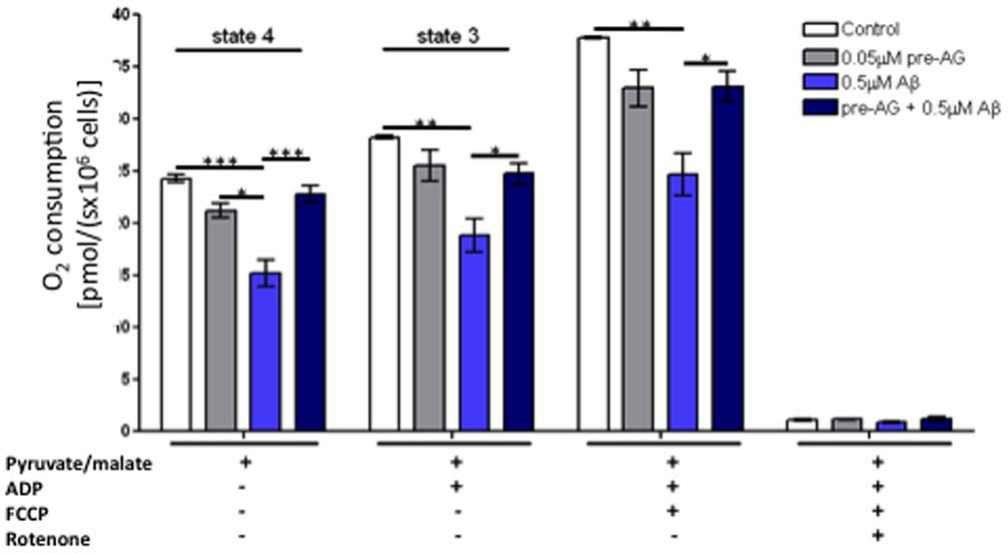
High-resolution respirometry revealed a reduction of oxygen consumption in Aβ42-treated cells that was restored after pre-treatment with AG18051. O_2_ flux and consumption by vital cells was measured after addition of different agents: pyruvate/glutamate, ADP, FCCP, rotenone. Two-way ANOVA revealed a significant difference between the cellular respiration of the cells treated either with vehicle, Aβ42 or AG18051 alone, or AG18051 plus Aβ42 (p<0.0001) (see scheme Fig. 3A). The respiratory rates of mitochondria were significantly reduced in Aβ42-treated cells compared to control (vehicle treated) cells and cells pre-treated with AG18051 (24 h) before exposure to Aβ42. Values represent the means ± S.E. from n = 3–5 independent measurements. Post-hoc Bonferroni's Multiple Comparison Test analysis for single experimental respiratory conditions: *, p<0.05; **, p<0.01; ***, p<0.001.

To investigate the protective effect of AG18051 against Aβ42 toxicity at the mitochondrial level, we used a high-resolution respiratory protocol that we have established previously [49]. Specifically, physiological substrate combinations were used to investigate mitochondrial function in SH-SY5Y cells (Fig. 6). We compared OXPHOS, i.e. the entire electron transport system (ETS) that is composed of the four mitochondrial enzymes (complex I–IV) and the F_1_F_0_ATP synthase, in cells treated with either vehicle, Aβ42, AG18051, as well as in cells that were pre-treated with AG18051 followed by exposure to Aβ42 (see scheme, Fig. 3A). We used the NADH generating substrates pyruvate and malate to determine state 4 respiration (Fig. 6). State 3 respiration measures the capacity of mitochondria to metabolize oxygen and the selected substrate in the presence of a defined amount of ADP, which is a substrate for the ATP synthase (complex V). State 4 respiration represents a “basal-coupled” rate of respiratory chain activity and reflects activities of respiratory chain complexes and proton leakage across the inner mitochondrial membrane. We observed significantly reduced state 3 and state 4 respirations in Aβ42-treated cells (Fig. 6). After uncoupling with FCCP, the respiratory rate increased in the absence of a proton gradient, which indicates the maximum capacity of electron transport chain. This maximum OXPHOS capacity was again significantly impaired in Aβ42-treated (Fig. 6).

Of note, AG18051 was able to significantly ameliorate the Aβ42-induced global failure of mitochondrial respiration, but by itself had no effect *per se* on oxygen consumption attaining levels comparable to vehicle treatment (Fig. 6).

### AG18051 prevents the increase in ROS levels caused by Aβ42

**Figure 7:**
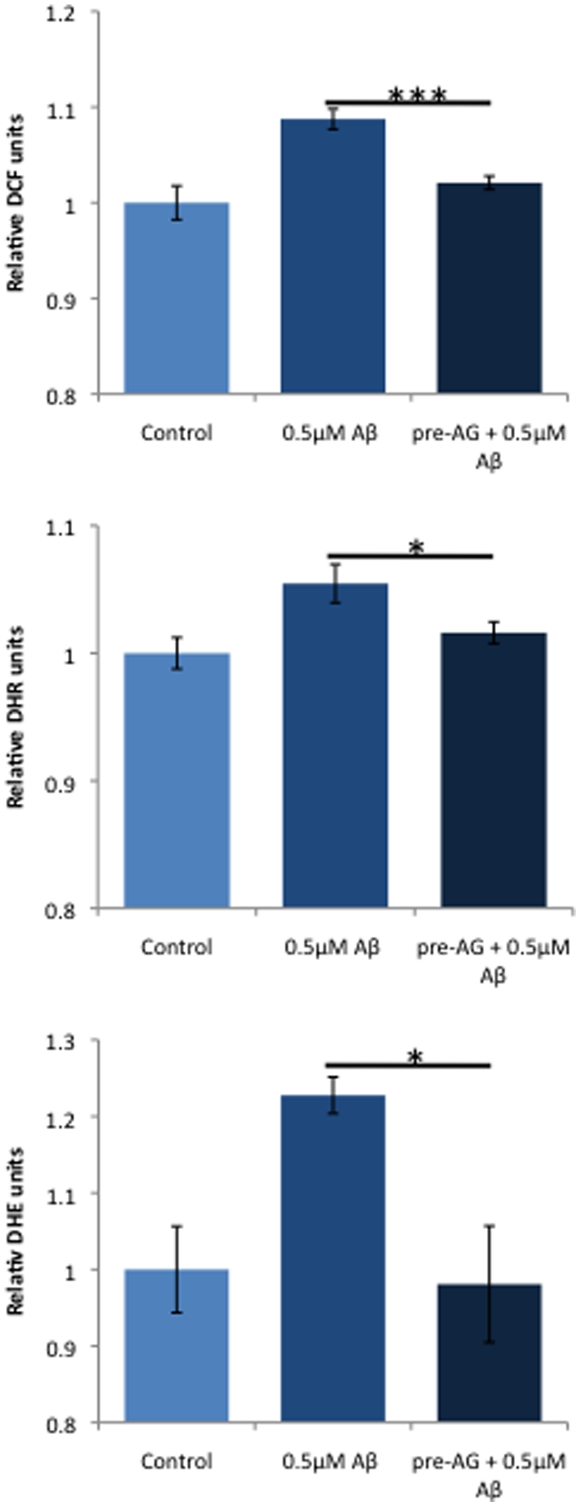
AG18051 pre-treatment prevents ROS generation induced by Aβ. Aβ causes reduced cellular (DCF) as well as mitochondrial ROS (DHR), e.g. reduced mitochondrial superoxide anion radicals (DHE). Levels are restored to vehicle upon pre-treatment with AG18051, irrespective of whether the SH-SY5Y cells have been incubated with Aβ or HA. *, *P*<0.05; ***, *P*<0.001.

Increased ROS levels have been implicated in AD [50], [51]. We had previously found that treatment of SH-SY5Y cells with Aβ42 caused significantly increased ROS (reactive oxygen species) levels, reflecting mitochondrial dysfunction and cell toxicity [52]. As pre-incubation with AG18051 was capable of preventing the toxicity induced by Aβ42, we pre-treated SH-SY5Y cells with AG18051 and exposed them then to Aβ42. Interestingly, cells pre-treated with 0.05–0.1 µM AG18051 were effectively protected from any Aβ42-induced ROS production, attaining levels comparable to vehicle treatment (Fig. 7). This suggests that AG18051 may prevent cell toxicity induced by Aβ42 in part by preventing the generation of ROS. In addition, the results also suggest that ABAD dysfunction may be upstream of ROS production since AG18051 can prevent ROS generation. Alternatively, AG18051 may trigger high estradiol levels, thereby counteracting ROS.

### AG18051 only partially protects against reductions in cell viability and estradiol levels in SH-SY5Y cells when deregulated by human amylin (HA)

**Figure 8:**
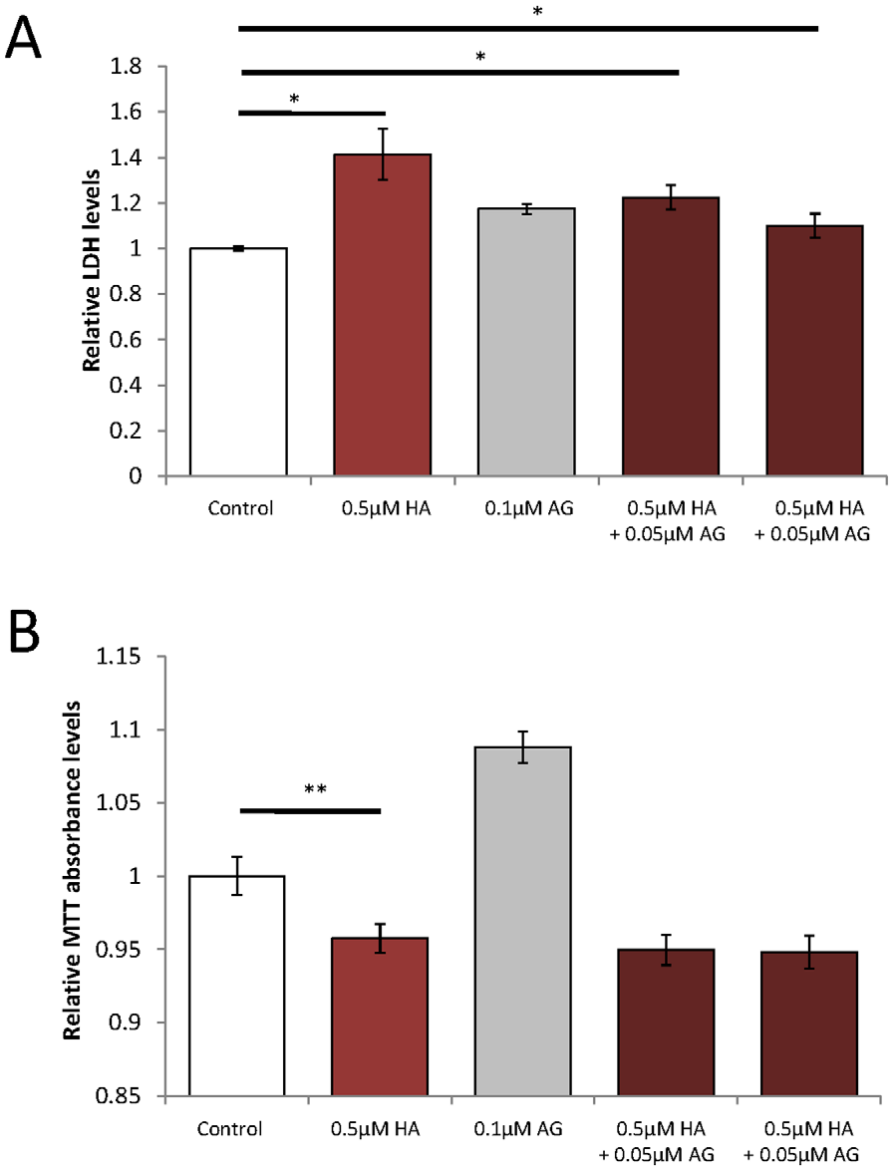
The ABAD inhibitor AG18051 partially prevents the toxicity of HA, but not its metabolic impairment. (A) Co-incubation of HA with AG18051 partially maintains levels of LDH release in SH-SY5Y cells suggesting that the toxicity of HA is partially mediated by ABAD. (B) Treatment with 0.5 µM HA significantly decreases metabolic activity as shown with the MTT assay, which is not prevented with co-incubation with AG18051. *, *P*<0.05; **, *P*<0.01.

Previously, we had found that Aβ42 and HA share toxicity pathways via deregulation of mitochondrial proteins [52]. However, as shown above, Aβ42 binds to and down-regulates ABAD activity, while HA fails to do so (Fig. 1). We nonetheless sought to determine whether AG18051 would have an effect in the toxicity assays used above to measure Aβ toxicity. We found that HA induced an increase in LDH levels revealing its toxicity (Fig. 8A), and a corresponding decrease in MTT absorbance revealing its effect on metabolic functions (Fig. 8B). Co-incubation of AG18051 with HA significantly prevented the increase in LDH levels when compared with HA alone, but did not fully maintain LDH to the levels of vehicle treatment indicating only a partial protection from HA-induced toxicity (Fig. 8A). Co-incubation of AG18051 with HA did not have any significant impact on MTT levels (Fig. 8B).

### AG18051 prevents the increase in ROS levels caused by Aβ42 and HA

**Figure 9:**
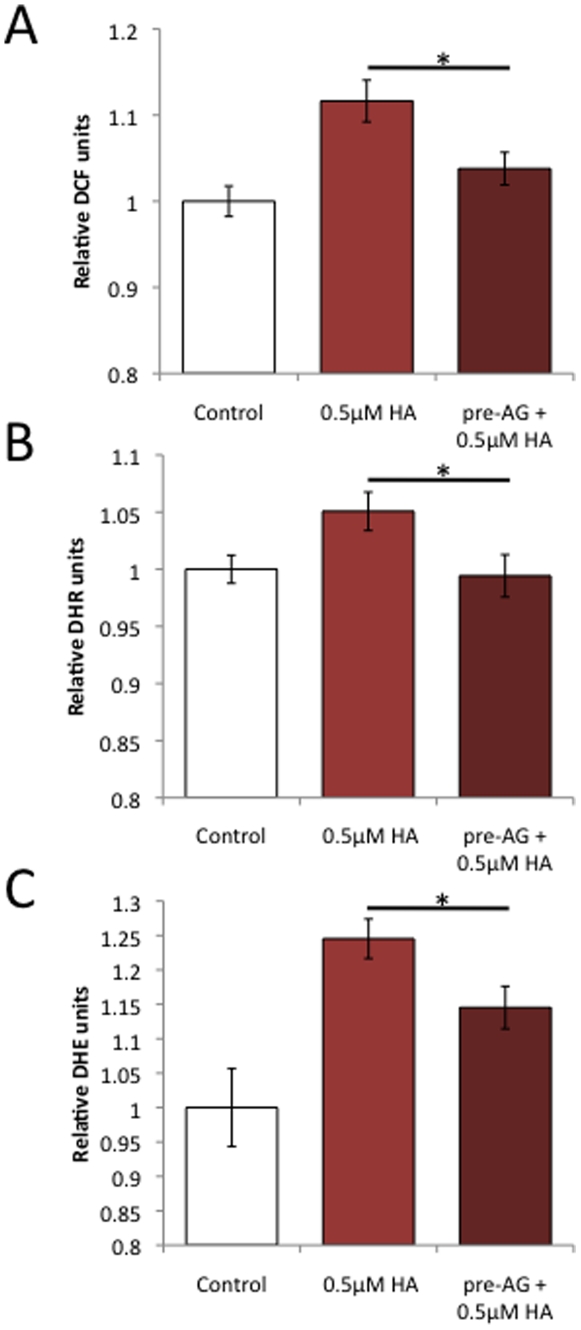
AG18051 pre-treatment prevents ROS generation also induced by HA. As for Aβ, HA causes reduced cellular (A) as well as mitochondrial ROS (B), e.g. reduced mitochondrial superoxide anion radicals (C). Levels are restored to vehicle upon pre-treatment with AG18051. *, *P*<0.05; ***, *P*<0.001.

Increased ROS levels have not only been implicated in AD but also T2DM [50], [51]. We had previously found that ROS levels in SH-SY5Y cells are significantly increased upon exposure to either Aβ42 or HA [52]. To determine if the limited protective effect seen by AG18051 on HA-induced toxicity may be due to an inhibition of ROS generation, we pre-incubated SH-SY5Y cells with AG18051 and then exposed them to HA. Similar to Aβ42 (Fig. 7), pre-treatment with 0.05 µM AG18051 completely prevented ROS generation by 0.5 µM HA (Fig. 9). Taken together with the ROS data obtained for Aβ42 (Fig. 7), this suggests that AG18051 may be neuroprotective by preventing ROS generation induced by either Aβ42 or HA.

## Discussion

Mitochondrial dysfunction has been recognized as a prominent, early event in AD, but the underlying mechanisms are only partly understood [53]. As a mediator of Aβ toxicity in AD, a role has been proposed for the mitochondrial protein ABAD, with evidence for a direct interaction of Aβ and ABAD [17]. Whether in the AD brain, intracellular Aβ - either in mitochondria or in the cytoplasm - is present at sufficiently high quantities to have a decisive role in disease is a matter to debate that has been revived by the recent analysis of Aβ plaque-forming 3xTg-AD using a panel of Aβ- and APP-specific antibodies [54]. Our current study however adds to the body of data revealing a role for ABAD in mediating Aβ, irrespective of its mode of interaction.

By using the novel compound inhibitor of ABAD, AG18051 [41], we revealed that Aβ-mediated toxicity, metabolic impairment and reductions in estradiol levels could be abrogated. However different from Aβ, the inhibitor only partially restored the toxicity of HA, that shares with Aβ its amyloidogenic properties, and it had no effect at all on the impaired metabolic activities or the reduced estradiol levels caused by HA. The compound though abolished both the Aβ42- and HA-induced increases in ROS levels. These effects may or may not require a direct interaction of Aβ and ABAD. Provided that Aβ enters mitochondria and binds ABAD, it is believed to gain access to the mitochondrial matrix via intracellular trafficking. Our pull-down experiments using a biotinylated preparation of Aβ reveal that this peptide binds to ABAD in a lysate, while in contrast, HA does not.

One of the established functions of ABAD is to convert in an equilibrium reaction estrone to estradiol, a known anti-oxidant in neuronal survival [31]–[33], [55]. Additional substrates are known for ABAD, such as the mitochondrial 2-methyl-3-hydroxybutyryl-CoA [40], [56].

ABAD is up-regulated in AD brain areas affected by Aβ pathology such as the cortex and hippocampus, as well as in Aβ-producing mouse models [17], [23], [26], [57]. In neuroblastoma cells, the cytotoxic effects of Aβ are enhanced by ABAD over-expression, and blocked with anti-ABAD antibodies [58]. Moreover, synthetic Aβ fragments have been shown to bind and inhibit ABAD *in vitro* [39]. As cells expressing catalytically inactive mutants of ABAD failed to show an enhanced sensitivity to Aβ, it has been suggested that it is the enzymatic activity that is required for mediating Aβ toxicity [38]. *In vivo*, ABAD over-expression potentiates the toxic effects of Aβ, and obliteration of Aβ-ABAD complexes restores cell viability and memory deficits in transgenic mice [17], [26]. This inhibition was achieved using a truncated version of ABAD as a decoy peptide (ABAD^DP^) [17], [26]. The authors of the study concluded that segregating ABAD from Aβ protects both mitochondria and neurons from Aβ toxicity by restoring ABAD's physiological functions.

Rather than employing a decoy peptide, we decided to use the small compound, AG18051, a novel ABAD inhibitor to investigate its putative protective effects. We performed co- and pre-incubation experiments to determine whether the restoration of estradiol levels by AG18051 is due to a direct inhibition of the Aβ42-ABAD interaction, or an indirect mechanism. By determining estradiol levels as a functional read-out of ABAD activity, we found that exposure to Aβ42 significantly decreased estradiol levels (Fig. 3B, C). We have been using SH-SY5Y cells, a well-established neuroblastoma cell line [43], to determine the effects of AG18051 on ABAD. We found that pre-treatment of SH-SY5Y cells with AG18051 before Aβ42 exposure (Fig. 5C) was sufficient to prevent the decrease in cell viability. This suggests that AG18051 is capable of blocking Aβ42 toxicity, possibly in part by directly binding to ABAD and preventing the Aβ42 toxicity mediated by ABAD.

To determine whether these changes are due to ABAD specifically, we performed co-incubation treatments of a novel ABAD inhibitor, AG18051, with Aβ42. We found that AG18051 effectively obliterated the toxic effects of Aβ42 as demonstrated by the restoration of LDH release and MTT absorbance to levels indistinguishable from the vehicle control. Furthermore, AG18051 restored estradiol levels upon down-regulation by Aβ42. This effect is seen both in the cell lysate and the medium (data not shown), suggesting that Aβ42 exerts its toxicity by interfering with intracellular levels of estradiol while also decreasing its secretion into the extracellular environment. Our data indicate that Aβ42-induced toxicity is mediated by ABAD in part via the deregulation of estradiol, which may contribute to cell toxicity.

To determine whether the change in estradiol levels is due to a direct blockage of Aβ42 by ABAD, we performed a pull-down using biotinylated Aβ42 in the presence of AG18051 (Fig. 4). We found that AG18051 significantly decreased ABAD binding to biotinylated Aβ42, suggesting that AG18051 may be neuroprotective, in part by disrupting the physical interaction of ABAD with Aβ42. However, as binding was not completely abolished, our results suggest that AG18051 may also act indirectly to prevent cell toxicity. This is supported by the fact that AG18051 is capable of preventing ROS production by HA (Fig. 9) even though HA does not seem to bind to ABAD (Fig. 1B).

To unravel the effects of Aβ42 and the involvement of ABAD on the mitochondrial respiratory capacity, we performed whole cell recording of total cellular respiration in SH-SY5Y cells (Fig. 6). Consistent with previous findings investigating the effect of a stable APP over-expression in SH-SY5Y cells gaining a chronic overproduction of Aβ within the low nanomolar range [49], we observed a comparable impairment of oxygen consumption rates in cells treated with Aβ42 species for 5 days. Of note, we present for the first time clear evidence that pre-treatment with AG18051 prevented SH-SY5Y cells from a decline in metabolic energy pathways induced by Aβ42. The capacity of mitochondria to re-phosphorylate ADP in state 3 is dependent on the degree of coupling. Thus, pre-treatment with AG18051 prevented the ETC (electronic transport chain) from Aβ42 toxicity and rescued the coupling state of mitochondria. Importantly, the comparison of the mitochondrial energetic capacity in cells treated with vehicle or AG18051 revealed a similar bioenergetic homeostasis indicating that the inhibitor, by itself, had no significant effect on respiration. These results corroborate our findings demonstrating an AG18051-induced prevention of ROS formation caused by Aβ42, which in turn impairs mitochondrial function.

That a defective or insufficient mitochondrial function might play a potentially pathogenic role in another epidemic disease, Type 2 diabetes mellitus (T2DM), has emerged in recent years [59], [60]. The association of diabetes with obesity and inactivity indicates an important, and potentially pathogenic, link between fuel and energy homeostasis and the emergence of metabolic disease. Given the central role for mitochondria in fuel utilization and energy production, mitochondrial dysfunction at the cellular level can impact whole-body metabolic homeostasis [61]. T2DM is characterized by HA deposition in the pancreas, and AD by Aβ deposition in the brain [59]. Aβ and HA share a common β-sheet secondary structure, a strong determinant in toxicity [62], [63]. Supporting this notion we have shown previously that Aβ and HA share a mitochondrial toxicity profile [52]. In the present study we found that HA caused toxicity and impaired metabolic functions. When we co-incubated HA with AG18051 using the same conditions as for Aβ42, AG18051 significantly decreased levels of LDH compared to just HA alone, suggesting that HA toxicity is in part mediated by ABAD (Fig. 8A). Interestingly, co-incubation of AG18051 with HA did not significantly change levels of MTT absorbance, an established assay of mitochondrial function (Fig. 8B) [64]. We have previously shown that to mitochondria, at equimolar concentration, HA is more toxic than Aβ42 [52]. This suggests that a higher concentration of AG18051 may be required to restore the HA-induced mitochondrial toxicity. Interestingly, while the MTT assay is a reliable test for metabolic impairment, it does not always precisely reflect neuroprotective effects, suggesting that the LDH assay is more accurate in determining neurotoxicity [65]. It is therefore possible that the neuroprotective effects of AG18051 against HA treatment differs from that of Aβ42. By extrapolation, this also means that HA and Aβ42 may exert a differential toxicity on ABAD. This is a possibility, as exposure to HA did not alter estradiol levels, different from Aβ42 (Fig. 1A).

Pre-treatment of SH-SY5Y cells with AG18051 prevented both the cellular and mitochondrial ROS formation induced by Aβ42 and HA suggesting that ABAD is involved in their mechanism of toxicity. We have shown previously that increased ROS generation and reduced mitochondrial complex IV activity was the common mechanism of toxicity of HA and Aβ42 [52]. This is in agreement, in the case of Aβ, with other reports showing that Aβ results in ROS generation and reduction of complex IV in AD mouse models and that there is a direct involvement of ABAD in these processes [17], [66], [67]. Now it appears that HA ROS generation is also mediated by ABAD. However, while AG18051 may be protecting against most of the toxic effects of Aβ42, it does so only partially for HA. This indicates that HA may have additional toxicity pathways unrelated to ROS generation or ABAD.

The mechanism of protection by AG18051 is probably through its inactivation of ABAD's catalytic activity. Inactive mutants of ABAD do not enhance the toxicity of Aβ that is observed when wildtype ABAD is over-expressed, despite the fact that Aβ still binds to the mutant ABAD with the same affinity as wild-type [68]. It has also been reported that siRNA-ABAD and the subsequent reduction of ABAD expression prevents the toxic effects of Aβ in SH-SY5Y cells induced to over-express ABAD by corticosterone and Aβ [67]. But why would the catalytic activity of ABAD be harmful when Aβ is bound to it? In view of the participation of ABAD in mitochondrial RNAse P, which is responsible for the centrally important processing of the mitochondrial ETC mRNA [37], one possibility is that ABAD's primary function is as an RNAse P and that Aβ and HA induce a toxic gain of function related to its catalytic activity compromising its RNAse P function. This would result in aberrant processing of the ETC mRNA, a dysfunctional ETC and ROS generation. Aβ may do this directly by binding to ABAD and, while there is no evidence that HA directly interacts with ABAD, our study shows that ABAD is nevertheless key to the toxicity of HA.

In conclusion, we extend previous findings on the role of mitochondria, and in particular the mitochondrial enzyme ABAD, in mediating Aβ42 toxicity. We established a neuroprotective effect of AG18051, a novel ABAD-specific inhibitor, and showed that it promotes cell survival in part by preventing the generation of ROS and stabilizing estradiol levels. Our findings extend previous studies suggesting ABAD activity as a suitable biomarker for impaired brain functions. We further present AG18051 and related compounds for consideration in therapeutic strategies targeting AD.
